# An injectable gambogic acid loaded nanocomposite hydrogel enhances antitumor effect by reshaping immunosuppressive tumor microenvironment

**DOI:** 10.1016/j.mtbio.2025.101611

**Published:** 2025-02-24

**Authors:** Dan Lei, Wanru Wang, Jianhang Zhao, Yingling Zhou, Ying Chen, Juanjuan Dai, Yuling Qiu, Haoyue Qi, Chunhua Li, Boyao Liang, Baorui Liu, Qin Wang, Rutian Li

**Affiliations:** a The Comprehensive Cancer Center, Nanjing Drum Tower Hospital, Clinical College of Nanjing Drum Tower Hospital, Nanjing University of Chinese Medicine, Nanjing, China; b State Key Laboratory of Organic Electronics and Information Displays & Institute of Advanced Materials (IAM), Nanjing University of Posts & Telecommunications, Nanjing, China; c The Comprehensive Cancer Centre of Nanjing Drum Tower Hospital, Affiliated Hospital of Medical School, Nanjing University, Nanjing, China; d State Key Laboratory of Analytical Chemistry for Life Science, China; e Medical School of Nanjing University, China

**Keywords:** Cancer immunotherapies, Gambogic acid loaded nanoparticles, Traditional Chinese medicine, Temperature responsive injectable hydrogel, Immunosuppressive tumor microenvironment remodeling

## Abstract

Gambogic acid(GA)is a natural compound that exhibits strong antitumor activity against a variety of tumors. However, its poor water solubility, low specificity, and high toxicity lead to inevitable systemic adverse effects. To minimize side effects, combining gambogic acid (GA) with delivery systems such as nanohydrogels to develop an in situ vaccine system (ISV) shows great promise. In this study, we loaded GA into a novel in situ nanocomposite hydrogel vaccine system (Gel-NPs@GA) along with a near-infrared (NIR) fluorescent dye, IR-1061. The Gel-NPs@GA system allowed for temperature-triggered gelation, simplifying injection and the in vivo formation of a drug-releasing gel, with near-infrared monitoring for drug metabolism. Slow, continuous release of gelatinase-targeted GA nanoparticles from the hydrogel occurs, followed by cleavage of mPEG-peptide-PCL conjugates by gelatinase, causing particle aggregation for endocytosis by tumor cells. This approach tackled solubility issues and curbs excessive GA release, boosting therapeutic drug levels. The sustained GA release induces tumor cell apoptosis, releasing tumor antigens and reprogramming the immune-suppressive tumor microenvironment. In the CT26 colorectal cancer mice model, this in situ vaccine system significantly inhibited tumor growth. By integrating information about immune cell clusters within the tumor microenvironment with RNA sequencing results, we hypothesized that Gel-NPs@GA could synergistically stimulate the immune response through various pathways, promote the maturation of dendritic cells (DCs), increase the infiltration of T cells, and thereby remodel the tumor's immune microenvironment.

## Graphical abstract

**Figure:**
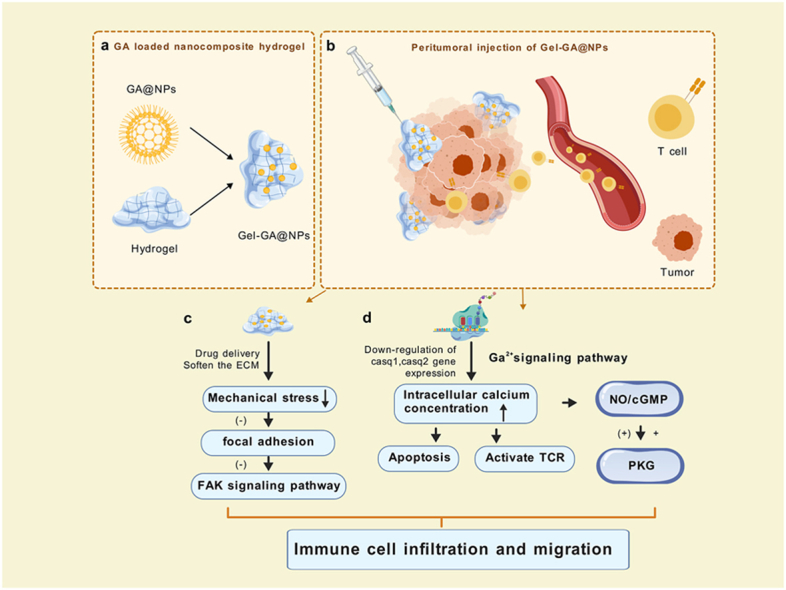

## 1 Introduction

ISV (in situ vaccine) is a specialized type of immunotherapy that aims to stimulate immune response directly within the tumor microenvironment. The fundamental concept required for successful therapeutic cancer vaccines is to deliver antigens and adjuvants to antigen-presenting cells (APCs), such as macrophages and dendritic cells (DCs), which subsequently induce antigen-specific T-cell responses [1]. By triggering an immune response at the site of the tumor, ISVs can promote immune system to recognize and eliminate cancer cells in injected tumor region, and can also inhibit the growth of metastatic tumors, which is socalled abscopal effect [2]. It has been reported that radiotherapy or intra-tumoral immune adjuvant injection can have an ISV effect that provide tumor antigens to activate antigen‐presenting cells (APCs) and initiate tumor-specific immune responses [3]. However it can trigger a significant inflammatory response, which may result in symptoms such as fever or allergies. Consequently, the choice of injection agents is of utmost importance [4]. Besides, strategies to prolong the stimulation time of antigens on immune cells can help to amplify the immunological response.

Traditional Chinese herbs constitute a valuable resource for the identification and development of novel cancer therapeutic strategies [5]. The antitumor efficacy of Chinese herbal medicines has been widely recognized [6]. GA, as an extract from traditional Chinese medicine, exhibits significant anti-cancer effects against various cancer types by targeting different molecular pathways to modulate immunity. GA not only can serves as an effective ingredient that directly kills tumor cells but also as an immunoadjuvant that enhances the infiltration of CD3^+^CD8^+^ T cells within tumor tissues by modulating the tumor immune microenvironment and inducing the maturation of DCs in draining lymph nodes [7]. Most importantly, GA has been approved by the CFDA to enter Phase II clinical trials [8], which also reflects that GA has great potential for application. However, its limitations of poor water solubility, toxicity, and low selectivity seriously hinder its clinical application.

Smart nanocomposite hydrogels offer unique advantages for local drug delivery, which can solve challenges, such as inadequate local drug concentration and rapid drug clearance [9]. Nanoparticles (NPs) can passive or active targeted accumulated to tumor and slowly release loaded drugs [10,11], which can help increase water solubility, reduce side effects and enhance antitumor efficiency. What's more, hydrogels are widely used in the field of biomedical engineering due to their capabilities of storing multiple drugs, protecting encapsulated contents for degradation, good biocompatibility, and flexibility [12]. Besides, their unique hydrated architecture closely resembles the extracellular matrix (ECM), providing a favorable microenvironment for cells and bioactive molecules. This environment allows immune cells, such as T cells and dendritic cells, to be stored long-term while maintaining their vitality [13]. Therefore, nanocomposite hydrogels exhibit a combination of the fundamental properties of both the NPs and the hydrogel, and serve as a promising delivery system for local drug administration [14].

Here, we established a novel in situ nanocomposite hydrogel vaccine system (Gel-NPs@GA) along with a near-infrared (NIR) fluorescent dye, IR-1061 for the treatment of CT26 colon cancer. The nanocomposite hydrogel was composed of PEG-pep-PCL gelatinase-responsive nanoparticles (NPs) and thermosensitive nanohydrogel. The hydrogel can quickly transform from an injectable flowing solution at low temperature into a nonflowing gel state when injected into tumors with normal body treatment. NPs could be fixed by the temperature-responsive gel to the tumor tissue and slowly released into the tumor region. After release from the gel, Gelatinase-cleavable peptide Pro-Val-Gly-Leu-Iso-Gly (PVGLIG) can be degraded by MMP2/9 in tumor tissue, which induced the aggregation of NPs, effectively taken up by cancer cells [15], improved GA concentration in tumor cells and enhanced the antitumor effect of GA. Antigens derived for apoptosis tumor cells can be uptake by DCs to stimulate T cell activation and active antitumor immune response. Finally, thanks to the favorable microenvironment of hydrogels, the tumor treated with Gel-NPs@GA showed the highest T and DC cell infiltration, offsetting the immunosuppression of TME.

## 2 Materials and methods

Cell culture medium and supplements were purchased from Gibco (NY, USA). IR-1061, Bovine serum albumin (BSA), and Hydroxypropyl cellulose (HPC aa) were purchased from Sigma-Aldrich Company (USA). Gambogic acid (GA) reagents were purchased from Yuanye Biology Company (Shanghai, China). Mouse antibodies were purchased from Biolegend (CA, USA). CT26 colon cancer cells were sourced from the Shanghai Institute of Biochemistry and Cell Biology Cell Bank and cultured in RPMI 1640 medium with 10 % FBS and 1 % PBS at 37 °C and 5 % CO_2_. Female Balb/c mice, 5–6 weeks old, were acquired from Shanghai Sippr-BK Laboratory Animal Co. Ltd. All animal experimental protocols were approved by the Laboratory Animal Care and Use Committee of the Affiliated Nanjing Drum Tower Hospital of Nanjing University Medical School (2022AE01011).

### 2.1 Preparation and characterization of NPs@GA and Gel-NPs@GA

The nanoparticle carrier mPEG-Pep-PCL was synthesized following the scheme that has been previously reported [15]. We prepared GA nanoparticles using the nano-precipitation method. Briefly,PEG-pep-PCL copolymers and GA were dissolved in THF(Tetrahydrofuran) in the appropriate proportions. Then, we injected the solution into the deionized water when the moment the deionized water was in high-speed vortex to ensure that the two liquids mixed well within milliseconds. Typically, the ultrasonic crusher was utilized to accomplish this task. The mixed solution was placed in a fume hood for at least 12 h for volatilization of the organic solvent. And then, the nonincorporated drugs were removed by dialysis (MWCO 0.45 μm). Finally, the solution was centrifuged at 15,000 rpm and 4 °C for 30 min to collect gambogic acid nanoparticles.

The particle size, zeta potential, and polydispersity index (PDI) of NPs@GA were measured using a Mastersizer 3000 laser diffraction particle size analyzer (Malvern Instruments Ltd, UK). The drug encapsulation efficiency of NPs@GA was determined using a multifunctional microplate reader by measuring ultraviolet absorbance at 360 nm.

The IR-1061-HPC-AA/BSA hydrogel was synthesized by dissolving BSA and IR-1061-HPC-AA in deionized water, resulting in separate solutions with 20 % mass fractions of BSA and IR-1061-HPC-AA. The prepared nanoprecipitates were added to the resulting solution and mixed thoroughly to obtain a solution of Gel-NPs@GA.

To assess the injectability of the hydrogel, we employed a single-needle injection system. The hydrogel was drawn into a 22 G syringe and expelled to evaluate its injectability. The self-healing ability of the Gel-NPs@GA was studied through tensile tests. Hydrogels containing IR-1061 and ICG dye were prepared and subsequently tested for their self-healing properties following attachment. To verify that the Gel-NPs@GA hydrogel can be applied in vivo, we used an IVIS spectral imaging system for near-infrared imaging and selected 5-week-old female Balb/c mice, injecting 150 μl Gel-NPs@GA hydrogel subcutaneously. After anesthetizing the mice with isoflurane, near-infrared imaging was performed at various time points using the imaging instrument.

### 2.2 In vitro evaluations

#### 2.2.1 Cell apoptosis

Apoptosis in CT26 cells was assessed via the Annexin V-FITC/PI assay. Cells were treated with NPs@GA at concentrations of 0, 25, and 50 μg/mL in triplicate. After collecting 1x10∗5 cells, they were washed with precooled saline and resuspended in 100 μL of Binding Buffer. Annexin V-FITC (5 μL) and PI Staining Solution (10 μL) were added, mixed gently, and incubated in the dark at room temperature for 15 min. Post-incubation, the mixture was diluted with 400 μL of Binding Buffer and kept on ice. Samples were analyzed within 1 h using a flow cytometer. After processing the cells according to the aforementioned method, we observed the fluorescence staining condition of tumor cells under a fluorescence microscope.

#### 2.2.2 Cell viability assay

Cells were seeded at a density of 3000 cells per well in a 96-well plate and incubated in a humidified environment at 37 °C with 5 % CO_2_ for 24 h. Subsequently, the cells were subjected to different concentrations of hydrogel components (0, 12.5, 25, 50, 100 μg/μL) for 24 h and 48 h. Following treatments, the CCK-8 solution was added to the medium to achieve a 10 % concentration, and the cells were further incubated for approximately 1h. Finally, the absorbance of the solution was measured at 450 nm using an enzyme-labeled instrument (Tecan Infinite F200).

CCK8 assays were also conducted to evaluate the viability of CT26 cells and MC38 cells treated with GA, NPs@GA, and Gel-NPs@GA.

#### 2.2.3 Cellular uptake assay

For the cellular uptake study, coumarin6 was chosen as a fluorescence probe to track the location of the nanoparticles. Coumarin6-loaded nanoparticles were obtained through the nanoprecipitation method and co-cultured with CT26 cells. After incubation for 24 h, the cells were rinsed twice with normal saline to remove excess coumarin 6 nanoparticles and imaged using a fluorescence microscope.

### 2.3 In vivo evaluations

#### 2.3.1 NIR imaging of Balb/c mice

NIR imaging was used to detect the sustained-release effect of NPs@CY7 in vivo when loaded in hydrogels. The Balb/c mice were anesthetized and scanned using the IVIS spectral imaging system at indicated time points (0, 1, 2, 4, 6, 8, 10, 24, 48, 96, 144, 168, 240 h) after intratumor injection with Gel-NPs@CY7, Gel-CY7, and free CY7 respectively.

#### 2.3.2 In vivo antitumor activity

To evaluate the anti-tumor effect, 2∗10^6^ CT26 cells in 100 μL PBS were subcutaneously injected on the right wing of each female Balb/c mice aged 6 weeks. After 6 days later, the tumor volume reached 75–100 mm^3^. The mice were randomly divided into 5 different treatment groups (n = 6): GA (5 mg/kg)、NPs@GA、Gel-NPs@GA、Gel、NS (control group), ensuring similar average tumor volumes. They received different treatments (100ul) peritumorally on day 7. Then the tumor volume and body weight of mice were recorded every two days until the 21st day and calculated tumor volume (V) using the formula: V = 0.5 × L × W^2^. Mice were sacrificed on day 21 and tumors were stripped and weighed.

#### 2.3.3 Antitumor immune response analysis

We obtained fresh tumors, lymph nodes, and spleen tissues for immune cell analysis via flow cytometry. Tumors were minced and incubated with type IV collagenase at 37 °C for 3 h. The suspensions were filtered and resuspended in PBS. All samples were stained with specific antibodies in the dark at 4 °C for 30 min, washed, and analyzed using Flow Jo. The monoclonal antibodies employed included CD3-FITC, CD4-PE/CY7, CD8-APC, CD11C-FITC, CD80-APC, and CD86-PE-CY7.

#### 2.3.4 RNA sequencing method for mouse CT26 tumor tissues

Tumor tissue samples were collected from the Gel-NPs@GA and NS groups. Freezable tubes were prepared for sample storage. Samples were transferred to pre-prepared freezer tubes using tweezers, rapidly cooled in liquid nitrogen, and stored at −80 °C. Library construction and RNA sequencing were performed by GENEWIZ (Suzhou, China). Transcriptome sequencing included RNA extraction, quality assessment, library preparation, purification, detection, quantification, cluster generation, and sequencing. Stringent quality control was maintained at each stage. After successful testing, libraries were combined based on effective concentration and target data volume, followed by Illumina sequencing.

### 2.4 In vivo safety evaluation

#### 2.4.1 Biosafety assessment

After 14 days of treatment, mice were sacrificed, and tumor tissues along with major organs (heart, liver, spleen, kidney, lung) were extracted for histological analysis. Tissues were fixed in 4 % formaldehyde, embedded in paraffin, sectioned, and stained with H&E.

#### 2.4.2 In vivo degradation of hydrogel

To assess hydrogel degradation in mice, we used 5-week-old female Balb/c mice, administering 150 μl hydrogel subcutaneously. One mouse was euthanized every 3 days for dissection of the injection site to observe hydrogel degradation and surrounding skin morphology, with findings documented via photography.

### 2.5 Statistical analysis

Statistical analyses were performed using GraphPad Prism 8.0.2 statistical software. All results were expressed as ‾X±SEM. P value was calculated by a two-tailed unpaired *t*-test or ANOVA. P < 0.05 meant that the difference was statistically significant (ns P > 0.05, ∗P < 0.05, ∗∗P < 0.01, ∗∗∗P < 0.001, ∗∗∗∗P < 0.0001). Flow cytometry data were analyzed using FlowJo software. Graphics were drawn using Bio-GDP and ImageJ software.

## 3 Results

### 3.1 Synthesis and characterization of NPs@GA

**Fig. 1:**
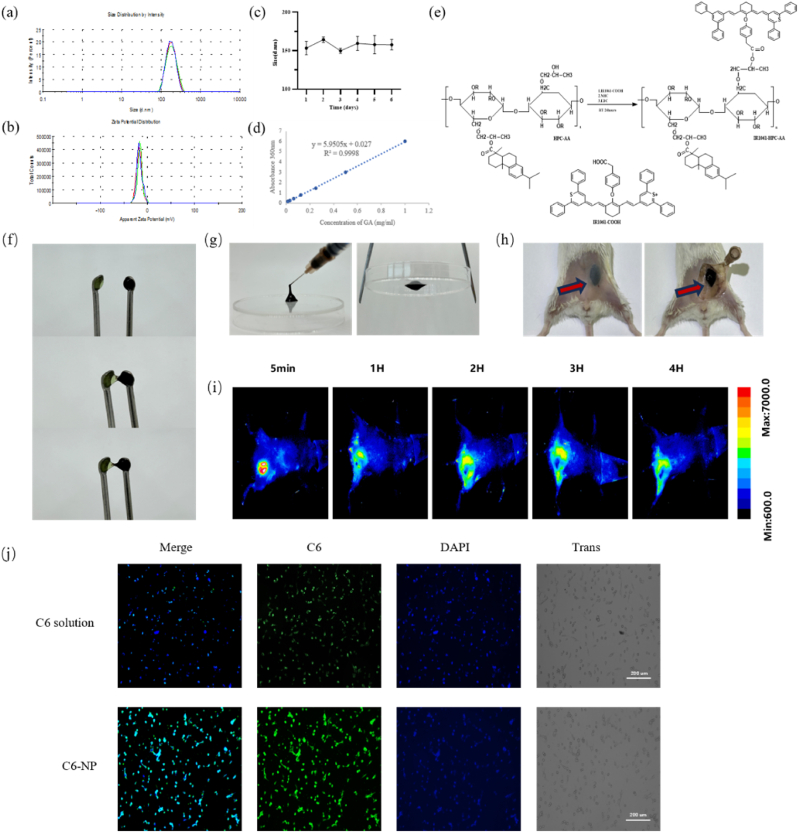
Characterization of NPs@GA and Gel-NPs@GA. (a) Size distribution of NPs@GA. (b) Zeta potential of NPs@GA. (c) Stability studies. (d) The linear relationship between the UV–vis absorbance and concentration of Gambogic Acid (360 nm). (e) The synthesis route of the hydrogel. (f–g) Evaluation of self-healing, injectability, and adhesion of hydrogels. (h) Evaluation of in vivo gel formation capacity. (i) In vivo fluorescence imaging with deep tissue penetration. (j) The fluorescence microscopy images of CT26 cells incubated with C6solution and C6 loaded-NPs for 24h.

Since gelatinase is ubiquitously expressed in malignant tumors, we have developed gelatinase-stimuli NPs by inserting the gelatinase-cleavable peptide Pro-Val-Gly-Leu-Iso-Gly (PVGLIG) into the linkage between polyethylene glycol (mPEG) and polycaprolactone (PCL) segments, resulting in the formation of mPEG-Pep-PCL NPs [16]. These 'smart' nanoparticles exhibit a passive tumor-targeting ability facilitated by microenvironment stimuli strategies. Gambogic acid is a hydrophobic herbal monomer, and we employed the aforementioned copolymer to encapsulate gambogic acid via the nanoprecipitation method, creating NP@GA for characterization (Fig. 1a–b). The initial average diameter of NPs@GA was 173.2 ± 1.19 nm (n = 3, SD ± SE) with a zeta potential of −21.7 ± 1.46 mV (average and standard deviation of 3 independently prepared batches) and polydispersity index of 0.057 ± 0.01 (n = 3, SD ± SE) at pH 7.4(Table. s1).

To assess the stability of NPs@GA, we placed the NPs in a 37 °C PBS bath and agitated them continuously for seven days. We measured the particle size at the same time every day. No significant changes were observed, indicating that NPs@GA exhibits good stability (Fig. 1c). The standard curve of GA was measured by adopting standard drug solutions with different concentrations (1-0.5-0.25-0.125-0.0625-0.03125-0.015625 mg/ml) respectively (Fig. 1d). And the drug loading capacity and encapsulation efficiency of NPs@GA was 25.31 ± 0.01 % and 83.05 ± 0.02 % respectively (Table. s2).

We further analyzed the peak retention time of GA and NPs@GA using high-performance liquid chromatography (HPLC). The results showed that both substances eluted for around 5 min, indicating that the composition of GA remained unchanged during nanoparticle preparation. Moreover, we placed NPs@GA of the same concentration in solutions with pH 7.5 and pH 3 to observe changes in the peak area of GA (Fig. s1). We noted a slight decrease in GA content in the acidic environment. According to the studies, the stability of GA may be slightly compromised under acidic conditions [17]. However, subsequent in vivo antitumor experiments have demonstrated that GA can still exert significant antitumor effects in the acidic tumor microenvironment.

As previously mentioned, the mPEG-peptide-PCL conjugates are susceptible to digestion by gelatinase, leading to the shedding of the mPEG moiety. Subsequently, the de-PEGylated NPs experience aggregation, morphing into larger particles. This structural transformation enables the particles to preferentially accumulate within the tumor microenvironment. Additionally, the aggregated particles internalized by tumor cells through endocytosis potentially enhance the rate of cellular uptake and augment the intracellular concentration of the anticancer payload. To verify that NPs@GA possess similar enzyme-targeting properties, we conducted in vitro enzyme-targeting experiments. Compared to the nanoparticle solution without gelatinase, the NPs@GA solution exhibited turbidity upon the addition of gelatinase, indicating that the nanoparticles deform and aggregate in response, gradually accumulating (Fig. s2). Therefore, we inferred that NPs@GA can effectively release drugs in specific tumor microenvironments.

To explore the uptake capabilities of NPs@GA, coumarin-6-labeled NPs (C6-NPs) were cocultured with CT26 cells, and their uptake motif was observed utilizing fluorescence staining microscopy. The fluorescence intensity of the C6-NPs group showed significantly higher fluorescence intensity in the cytoplasm compared to the free coumarin-6 group (Fig. 1j). It is well-established that free small molecules traverse cellular membranes via passive diffusion, while NPs are internalized through endocytosis [18,19]. The progressive increase in NPs uptake within the cytoplasm could potentially amplify the anticancer effects of GA-NPs.

### 3.2 Synthesis and characterization of hydrogel

For constructing optimized HPC for the temperature-induced gelation, we coupled the hydrophobic abietic moieties on the HPC chains (HPC-AA) to achieve its LCST (Lower critical solution temperature) approach to the human body temperature of 37 °C. The synthesized HPC-AA solution was mixed with the coupling solution of IR-1061-conjugated BSA (10 wt%) to obtain the HPC-AA/BSA hydrogel solution (Fig. 1e). The ratio of HPC-AA solution to BSA solution significantly influences the formation of the gel system. In vitro study demonstrated that the 5:5 ratio (10 % HPC-AA solution: 10 % BSA solution) can rapidly form a gel within 4 min in a 37 °C water bath, exhibiting optimal viscoelastic properties(Fig. s3). At the 5:5 ratio, the loss modulus (G″) and storage modulus (G′) intersected first, achieving an optimal storage modulus after entering the plateau phase. The hydrogel assumed a solid-state gel behavior once the storage modulus surpassed the loss modulus (G' > G″)(Fig. s4) [20]. After determining the optimal solution ratio, the prepared NPs@GA was incorporated into the HPC-AA/BSA with a vortex mixer homogenizing the NPs in the solution, and the pH was adjusted to approximately 3 using dilute hydrochloric acid. The resulting solution was subjected to gelation transformation under heating in a water bath at 37 °C in vitro. We prepared two different colored gelatinous hydrogels with methylene blue dye and with IR-1061 in order to test the self-healing property of the nanocomposite hydrogel (Fig. 1f). The separated nanocomposite hydrogels could adhere to each other which indicated that the Gel-NPs@GA had self-healing potential. After that, we loaded the Gel-NPs@GA into a 22 G syringe as shown in (Fig. 1g), and confirmed that the nanocomposite hydrogel could be injected through the syringe while maintaining its normal form. When we injected the Gel-NPs@GA solution into mice, we found that a well-formed nanocomposite hydrogel could be established within the mice (Fig. 1h).

The second near-infrared (NIR-II) fluorescence imaging (FI) has received increasing attention owing to its capacity for precise diagnosis and real-time monitoring of the therapeutic effects [21]. The use of IR-1061 in the hydrogel can achieve in vivo fluorescence imaging with deep tissue penetration which provides a monitoring function for drug metabolism more conveniently (Fig. 1i).

### 3.3 In vitro anti-tumor experiments and cellular uptake experiment

**Fig. 2:**
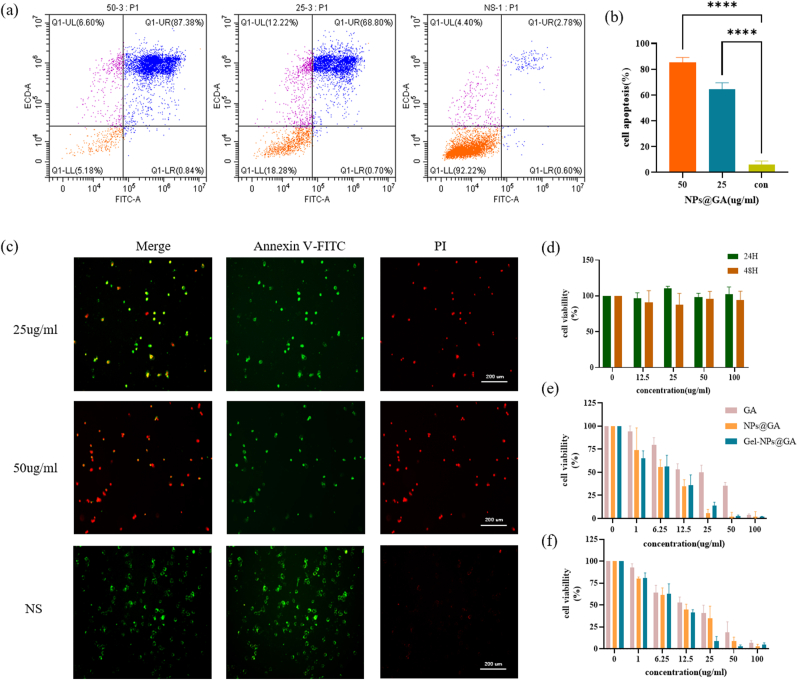
Nanoparticle-induced apoptosis and cellular uptake and cytocide effect by different treatments in vitro. (a) Representative images for flow cytometry of apoptosis in CT26 cells treated with different concentration of NPs@GA. (b) Statistical graph of apoptosis in CT26 cells treated with different concentration of NPs@GA. (c)The representative fluorescent images of apoptosis in CT26 cells by treatment of different concentration of NPs@GA. Green fluorescence represents live cells treated with annexin V-FITC and red fluorescence represents dead cells treated with PI. Scale bar: 50 μm. (d) In vitro cytotoxicity of different concentrations of HPC-AA/BSA hydrogel acting on CT26 cells for 24 h and 48 h. (e–f) In vitro cytotoxicity of different concentrations of Gel-NPs@GA, NPs@GA and GA acting on CT26 and MC38 cells for 24h. (For interpretation of the references to color in this figure legend, the reader is referred to the Web version of this article.)

Previous studies reported that GA has anticancer activity and is a potent apoptosis inducer [19,20]. We validated the apoptotic effects of NPs@GA through flow cytometry experiments (Fig. 2a), and confirmed it via fluorescence staining microscopy (Fig. 2b). Moreover, we found that the tumor cell apoptosis induced by GA showed a dose-dependent manner, and NPs@GA possess superior capacity to induce tumor cell apoptosis than free GA. Whereas HPC-AA/BSA hydrogel had little toxicity to tumor cells, achieving approximately 97 % cell viability at concentrations up to 100 μg/ml after co-incubation with tumor cells for both 24 h and 48 h (Fig. 2d). To demonstrate that Gel-NPs@GA exhibit superior synergistic therapeutic effects in vitro, we examined over a 24-h period (Fig. 2e–f). The cytotoxicity of Gel-NPs@GA against CT26 and MC38 cells after 24 h indicates significantly higher antitumor activity compared to the free drug. In contrast to the GA and NPs@GA treatment groups, the percentage of viable cells in the Gel-NPs@GA-treated group diminished notably. We also employed the Calcein-AM/PI staining kit to assess drug-induced cytotoxicity in CT26 cells. Fluorescence microscopy provided clearer insights into the cytotoxic effects of different groups on tumor cells. According to the staining results (Fig. s5), the NS group exhibited the highest levels of green fluorescence. In contrast, both NPs, GA, and Gel-NPs@GA displayed a significant number of cells exhibiting red fluorescence, with Gel-NPs@GA showing the highest red fluorescence and lowest green fluorescence. This indicates that this nanocomposite hydrogel can enhance the antitumor capacity of GA.

### 3.4 Local Gel-NPs@GA treatment inhibited CRC growth

**Fig. 3:**
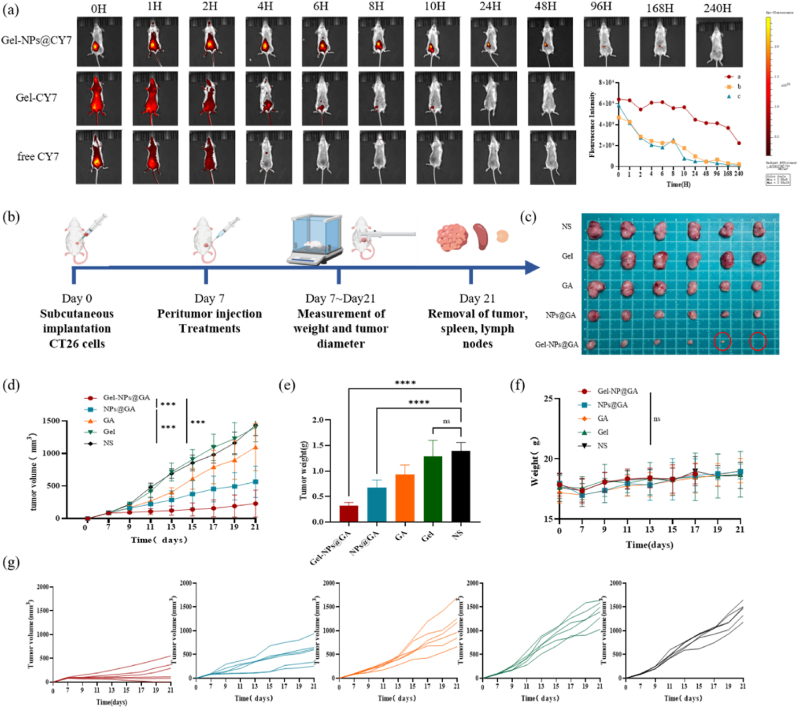
In vivo antitumor efficacy. (a) The mean fluorescence intensity of CT26 tumor-bearing mice injected by different treatments. (Gel-NPs@CY7: Nanocomposite hydrogels loaded with CY7; Gel-CY7: single hydrogels loaded with free CY7; free CY7) (b) Treatment scheme of peritumor injection with Gel-NPs@GA, NPs@GA, GA, Gel and NS CT26 tumor model. Balb/C mice were inoculated with CT26 cells subcutaneously on the left groin. When the tumors reached a size of 75–100 mm3, mice were injected peritumorally with the following drugs (n = 6): Gel-NPs@GA, NPs@GA, GA, Gel and NS. Mouse body weights and tumor volumes were measured every two days during the 14-day period after drug administration. On day 14 after treatment, tumors, spleens, and lymph nodes were removed from mice. (c)Tumor growth curves representing the average tumor volume of each group (n = 6). (d)Representative photos of the Isolated tumors with different treatments. (e)Tumor growth curves of each group. (f–g) Weight curves of isolated tumors and mice.

To investigate the drug release capacity of nano-composite hydrogels in vivo, we utilized CY7 fluorescent dye, which has a molecular weight similar to that of gambogic acid, as the released drug. In alignment with the synthesis method of NPs@GA, we first synthesized NPs@CY7 and then encapsulated the nanoparticles into HPC-AA/BSA hydrogel. Meanwhile, we set free CY7-loaded hydrogel and free CY7 as control groups. We employed the IVIS spectral imaging system for near-infrared imaging. As shown in Fig. 3a, the fluorescence intensity gradually decreased after being injected into the mice. And Gel-NPs@CY7 group showed a much stronger and prolonged fluorescence signal in tumor nodules compared to the Gel@CY7 group and CY7 group. This demonstrates that our drug delivery system can maintain localized drug release for up to ten days. Such a characteristic not only reduces the risk of off-target drug exposure but also diminishes the frequency of required doses over time.

Subsequently, we investigated the anti-tumor efficacy of Gel-NPs@GA. We generated a CT26 subcutaneous tumor-bearing model. Initially, we demonstrated that the NPs@GA treatment was significantly more effective in suppressing tumor progression compared to NS and Gel treatment. Notably, Gel-NPs@GA exhibited superior antitumor effectiveness compared to NPs@GA(Fig. 3d). Upon photographing the excised tumors(Fig. 3c–e), we noted that one mouse in the Gel-NPs@GA group had a reduced tumor volume compared to baseline, indicating a partial response (PR). Another mouse showed no detectable tumor, demonstrating a complete response (CR). These findings confirm the substantial anti-tumor efficacy of Gel-NPs@GA. Besides, none of the experimental groups exhibited any changes in body weight, which demonstrated the safety of Gel-NPs@GA in vivo shown in Fig. 3f.

### 3.5 Immune response induced by Gel-NPs@GA

**Fig. 4:**
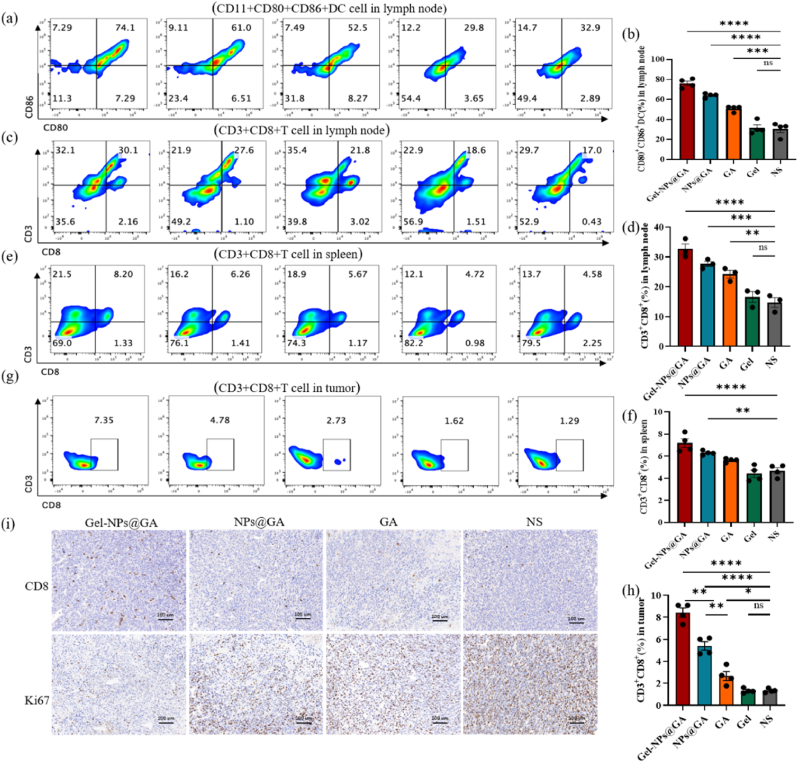
Generation of a systemic antitumor immune response by Gel-NPs@GA treatment(n = 4). (a) Typical images and percentages of activated dendritic cells in lymph node. (b) Percentage of activated dendritic cells (CD11C^+^CD80^+^CD86^+^) in lymph node. (c, e, g) Typical images and percentages of CD3^+^CD8^+^ T cells in lymph node, spleen and tumor. (d, f, h) Percentage of CD3^+^CD8^+^ T cells in lymph nodes, spleen and tumor. Data are presented as mean ± SD. ∗p < 0.05, ∗∗p < 0.01, ∗∗∗p < 0.001. (i) Representative images of CD8^+^ immunochemistry staining of tumors. Ki-67 immunochemistry staining of tumors.

To assess the immune response triggered by Gel-NPs@GA in vivo, we conducted the aforementioned experiment again and euthanized the mice 14 days post-treatment, subsequent to which we harvested the inguinal lymph nodes, spleens, and tumors to assess the immune response via flow cytometry. There was no difference between the NS group and the Gel group in almost all of the subsequent immune response tests. In the inguinal lymph nodes, the proportion of mDCs (CD11c^+^ CD80^+^CD86^+^) in the three groups (Gel-NPs@GA, NPs@GA, GA) increased as shown in Fig. 4a and b. The Gel-NPs@GA group exhibited the highest proportion of mature DCs (74.1 %), followed by NPs@GA and GA, both significantly surpassing the control group (32.9 %) (Fig. 4a). The results showed that the injection of Gel-NPs@GA increased the proportion of mDCs (CD11C ^+^CD80^+^CD86^+^) in TDLNs (Fig. 4b). We speculated that GA can induce tumor cell apoptosis, release antigens, promote the maturation of DC cells, and enhance antigen presentation effects. Meanwhile, Gel-NPs@GA can increase the concentration of GA within the tumor due to the storage capacity of hydrogels which can also make the immunocompetent cells such as DCs and T cells survive in the hydrogels for a long time without losing immune function. Thereby, Gel-NPs@GA exhibited strong activating effects on DCs due to its unique structure. Dendritic cells represent the most potent professional antigen-presenting cells (APCs) within the body, demonstrating formidable capabilities such as antigen capture and processing. Mature DC cells can uptake and process antigens, thereby activating naive T cells and further inducing their proliferation and differentiation into effector T cells [22,23]. The effector T cells (CD3^+^CD8^+^) play a direct role in tumor eradication, essential for immune defense against intracellular pathogens and tumor surveillance [24,25]. Additionally, both Gel-NPs@GA and NPs@GA groups demonstrated higher CD3^+^CD8^+^ T cell levels in lymph node and spleen (Fig. 4c–f). Further analysis of the immune response within the tumors revealed that the percentages of CD3^+^CD8^+^ T cells had notably risen in the Gel-NPs@GA, NPs@GA, and GA groups, as compared to the NS and Gel (Fig. 4g and h). Among these groups, the Gel-NPs@GA exhibited a markedly higher proportion of CD3^+^CD8^+^ T cells than that of other experimental groups.

These findings underscore the capacity of nanocomposite hydrogel treatment strategies to activate T lymphocytes and enhance the efficacy of antitumor immunotherapy. The data presented here elucidate the reasons behind the superior tumor-inhibiting efficacy observed in the Gel-NPs@GA group compared to the other three groups (NPs@GA, GA, Gel) [1]: Gel-NPs@GA that Benefited from the encapsulation effect of the hydrogel drug delivery system, allowed for a slow sustained release of NPs@GA which increase the concentration of GA within the tumor. Moreover Gel-NPs@GA can also make the immunocompetent cells such as DCs and T cells survive in the hydrogels for a long time without losing immune function [2]; The NPs@GA formulation, on the other hand, offered a combination of antitumor agents delivered to the tumor region with precision, bypassing the hydrogel's slow release mechanism and the specific structural requirements for DC targeting [3]; GA in the GA group was prone to rapid degradation within the circulatory system, leading to insufficient drug concentration at the tumor site—a condition that precluded the generation of a robust immune response due to a lack of tumor-specific antigens [4]; Blank hydrogels were not effective in killing CT26 tumor cells. These findings reflect the critical role of nanocomposite hydrogel carriers in optimizing drug delivery and modulating the tumor microenvironment for effective cancer therapy. Furthermore, the histologic analysis was performed in the tumor sections of mice. As provided in (Fig. 4 i), the proportion of CD3^+^CD8^+^ T cells in tumors treated with Gel-NPs@GA was significantly greater than in other treatment groups, aligning with the aforementioned flow cytometry results. The in-situ Ki-67 staining exhibited a reduction of cell proliferation after treatment with different groups respectively, especially for Gel-NPs@GA administration. In summary, the results suggested that composite hydrogel-based drug delivery strategies could induce a strong innate and adaptive antitumor immune response and that both DC and T cells were important.

### 3.6 RNA sequencing results from mice with CT26 tumor

**Fig. 5:**
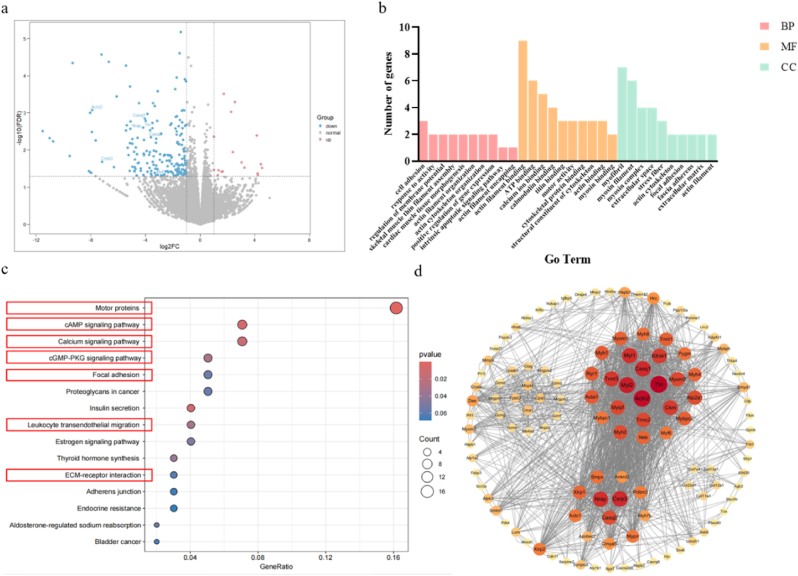
RNA-sequencing analysis of differential gene expression in tumors of CT26-bearing mice after Gel-NPs@GA treatment. (a) The distribution of differential transcripts (Gel-NPs@GA vs. Control). Abscissa: log2 for the fold change value, the larger the absolute value of the abscissa, the greater the expression difference multiple. Ordinate: Take -log10 for the p-value, the larger the ordinate value, the more significant the expression difference. Red dot: upregulated gene; blue dot: downregulated gene; gray dot: non-significant difference gene. (b–c) GO enrichment analysis of the DEGs involved in the biological effects induced by Gel-NPs@GA (Biological processes (BP), molecular functions (MF), and cell components (CC)). (d) Functional interaction networks of the DEGs. (For interpretation of the references to color in this figure legend, the reader is referred to the Web version of this article.)

To further explore the mechanism of Gel-NPs@GA in the tumor microenvironment, we performed high-throughput transcriptome sequencing on two groups of mice treated with Gel-NPs@GA and NS, respectively. The analysis revealed 17 up-regulated and 189 down-regulated transcripts (Fig. 5a). We conducted GO enrichment analysis on the DEGs, categorizing them into biological process (BP), molecular function (MF), and cellular component (CC). The top 10 enriched DEGs from each category include those related to cell adhesion pathway, adhesion plaques, actin, calcium channels, regulation of cell migration, and extracellular matrix (Fig. 5b). The pathways enriched by differentially expressed proteins based on KEGG analysis between Gel-NPs@GA and CON treatment groups primarily focus on motor protein, cAMP signaling pathway, calcium signaling pathway, cGMP-PKG signaling pathway, focal adhesion, leukocyte transendothelial migration and ECM-receptor interaction(Fig. 5c). To explore interactions among the DEGs, we constructed a PPI network using the STRING database, yielding four subnetworks and identifying seven hub genes: Ttn, Actn2, Myl2, Casq1, Casq2, Csrp3 and Nrap (Fig. 5d). These genes are involved in cell migration, calcium regulation, which is crucial for activating the tumor immune microenvironment. For example, Casq1 and Casq2 which are known for their role in calcium regulation, play a critical role in cell migration [26]. Gel-NPs@GA may enhance intracellular calcium ion concentration through calcium signaling pathways and downregulation of calsequestrin proteins (Casq1, Casq2). Compared to the control group, the Gel-NPs@GA group exhibited a significant downregulation in the expression levels of Casq1 and Casq2 proteins. Furthermore, KEGG pathway enrichment results revealed the activation of the cGMP-PKG signaling pathway which implies an association between Casq1, Casq2 induced calcium regulation and cGMP-PKG signaling pathway activation. The increased calcium ion concentration supports the anti-proliferative and pro-apoptotic effects of the NO/cGMP-PKG signaling pathway [27]. Thus, we hypothesized that Gel-NPs@GA down-regulated Casq1 and Casq2 proteins, increased calcium ion concentration, and subsequent activated NO/cGMP-PKG signaling pathway. Moreover, elevated calcium concentrations cluster around TCR can further enhance TCR activation and improve the ability to eliminate tumor cells [28]. Besides, the local delivery of hydrogel can soften the extracellular matrix, effectively alleviating the mechanical stress on tumor cells and downregulating mechanic transduction [29]. This stress can be regulated through the focal adhesion-FAK signaling pathway. FAK inhibitors have shown promising anti-tumor effects in clinical settings by inhibiting CAF activation, reducing collagen deposition, and further promoting the infiltration of anti-tumor immune cells, including CD8^+^ T cells and dendritic cells into the tumor, thereby remodeling the tumor microenvironment (TME) [30], which aligns with our in vivo flow cytometry results (Fig. 7).

To sum up, the Gel-NPs@GA treatment groups exhibited a notable increase in the expression of cell migration signaling pathways and genes associated with cell migration, thereby enhancing their antitumor effects in CT26 colon cancer mouse models. The observed upregulation of various immune cell subsets, as detected by flow cytometry, correlates with these findings, suggesting a highly responsive immune environment marked by the collaborative efforts of various immune cell types and signaling pathways. These outcomes may inform the creation of novel strategies for cancer immunotherapy interventions.

### 3.7 Biosafety assessment

**Fig. 6:**
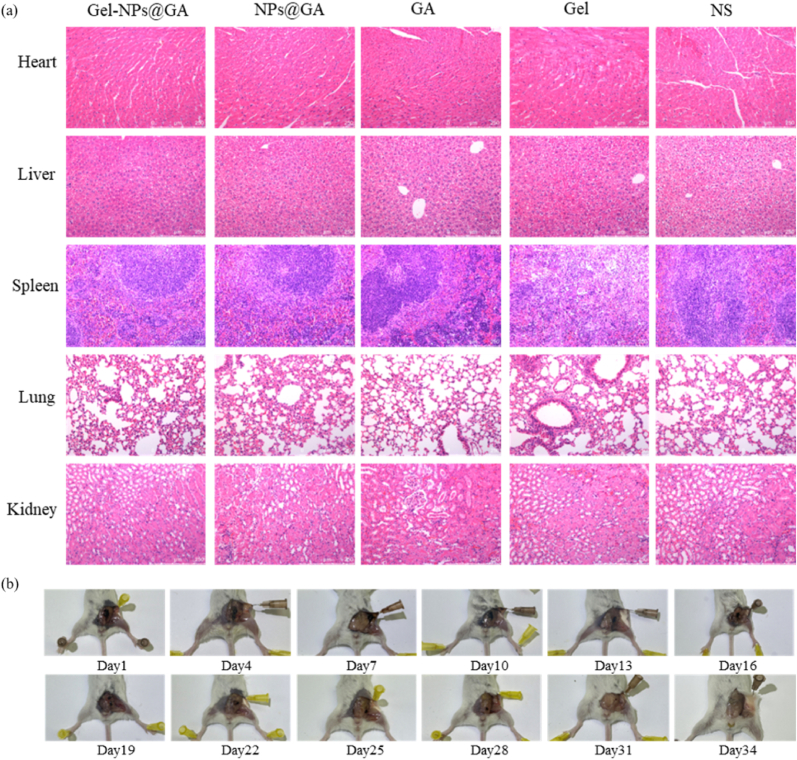
(a) Biosafety Assessment. Representative H&E-stained images of the heart, liver, spleen, lungs and kidneys. (b) In vivo degradation detection of Gel-NPs@GA.

To evaluate the biosafety of the drug delivery system, CT26 tumor-bearing mice were sacrificed 2 weeks after different treatments, and main organs (heart, liver, spleen, lungs, and kidneys) were collected for hematoxylin and eosin(H&E) staining. As shown in (Fig. 6a), no significant difference was observed in H&E-stained images among the Gel-NPs@GA, NPs@GA, GA, Gel, and NS groups indicating that the nanocomposite hydrogel system did not increase toxicity compared to a single system. Instead, it may reduce adverse reactions to other organs caused by the inability of a single drug to precisely target lesions. Previous studies have reported that gambogic acid has a short circulation time in the bloodstream, exhibiting toxic effects on myocardial cells and potentially impacting fetal development. Therefore, the local injection of nanocomposite gels loaded with gambogic acid in the form of in situ vaccines can significantly mitigate these side effects nanocomposite gels loaded with gambogic acid in the form of in situ vaccines can significantly mitigate these side effects.

**Fig. 7:**
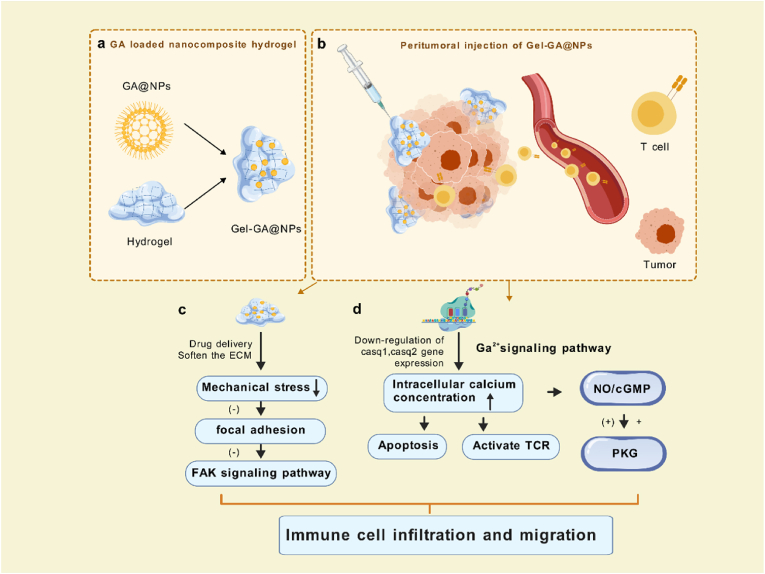
Schematic images of nanocomposite hydrogel preparation process and antitumor mechanism (Created with biogdp.com).

Furthermore, the successful degradation of hydrogels after injection into mice is a crucial factor affecting biocompatibility. We visually assessed their degradation in vivo for clarity. As shown in Fig. 6b, the hydrogels' volume decreased over time, fully degrading by day 34. No pathological changes, such as swelling or ulcers, occurred at the injection site. The hydrogels exhibited good safety and biocompatibility in mice.

## 4 Conclusion

In this study, we reported an injectable in-situ nanocomposite hydrogel vaccination system (Gel-NPs@GA) designed for treating colorectal cancer. The system allowed for targeted delivery and controlled release of drugs in the most minimally invasive manner through in situ injection for the purpose of visualizing drug metabolism and prolonging drug retention. In vivo experiments demonstrated that the in-situ vaccine (ISV) containing gambogic acid markedly inhibited tumor growth.

Mechanistically, we hypothesized that Gel-NPs@GA could suppress tumor proliferation and metastasis via multiple pathways. This is primarily attributed to the application of the ISV system technology, which effectively reverses the tumor immunosuppressive microenvironment. First, the targeted delivery and sustained release of gambogic acid promote apoptosis in tumor cells, thereby releasing tumor-associated antigens and pro-inflammatory cytokines, enhancing tumor immunogenicity. Second, the DEGs results indicated that the mice treated with Gel-NPs@GA reduced mechanical stress on cells due to the unique texture of the drug delivery platform, inhibiting further tumor cell deterioration and promoting the infiltration of anti-tumor immune cells by modulating the FAK signaling pathway. Additionally, based on the downregulation of hub genes expression and signaling pathway enrichment results, we speculated that the calcium signaling pathway/cGMP/PKG may also be regulated due to drug intervention. In summary, multiple pathways, through synergistic interactions, promote the maturation of antigen-presenting cells and activate effector T cells, effectively reversing the immunosuppressive tumor microenvironment. The construction of Gel-NPs@GA offers a safe and effective strategy with great potential for colorectal cancer (CRC) therapy.

## CRediT authorship contribution statement

**Dan Lei:** Writing – review & editing, Writing – original draft, Validation, Methodology, Investigation, Data curation, Conceptualization. **Wanru Wang:** Supervision, Methodology. **Jianhang Zhao:** Resources, Methodology. **Yingling Zhou:** Supervision. **Ying Chen:** Resources, Methodology. **Juanjuan Dai:** Supervision. **Yuling Qiu:** Supervision, Formal analysis. **Haoyue Qi:** Supervision. **Chunhua Li:** Methodology. **Boyao Liang:** Supervision. **Baorui Liu:** Resources, Project administration. **Qin Wang:** Writing – review & editing, Resources, Funding acquisition. **Rutian Li:** Resources, Project administration, Funding acquisition.

## Funding

This study was supported by National Natural Science Foundation of China (82272852), National Natural Science Foundation of China (82103687), Nanjing Medical Science and Technology Development Foundation (No. YKK23077), and Nanjing Medical Science and Technology Development Foundation (No. YKK22086).

## Declaration of competing interest

The authors declare that they have no known competing financial interests or personal relationships that could have appeared to influence the work reported in this paper.

## Data Availability

Data will be made available on request.
