# Supplementary material for: An injectable gambogic acid loaded nanocomposite hydrogel enhances antitumor effect by reshaping immunosuppressive tumor microenvironment
Source: Mater Today Bio. 2025 Feb 24;31:101611. doi: 10.1016/j.mtbio.2025.101611 (PMC11919334; doi:10.1016/j.mtbio.2025.101611)
Supplement: Multimedia component 1 [file mmc1.docx]

**supplementary information**

**An In****jectable Gambogic Acid Loaded** **Nanocomposite Hydrogel Enhances Antitumor Effect by Reshaping Immunosuppressive Tumor Microenvironment**

Dan Lei ^a^, Wanru Wang ^a^, Jianhang Zhao ^b^, Yingling Zhou ^a^, Ying Chen^b^, Juanjuan Dai ^c^, Yuling Qiu ^c^, Haoyue Qi ^c^, Chunhua Li ^a^, Boyao Liang^e^，Baorui Liu ^a,c,d***^, Qin Wang ^c,d**^, Rutian Li ^a,c,d*^

a. The Comprehensive Cancer Center, Nanjing Drum Tower Hospital, Clinical College of Nanjing Drum Tower Hospital, Nanjing University of Chinese Medicine, Nanjing China.

b. State Key Laboratory of Organic Electronics and Information Displays & Institute of Advanced Materials (IAM), Nanjing University of Posts & Telecommunications, Nanjing China.

c. The Comprehensive Cancer Centre of Nanjing Drum Tower Hospital, Affiliated Hospital of Medical School, Nanjing University, Nanjing, China.

d. State Key Laboratory of Analytical Chemistry for Life Science.

e. Medical School of Nanjing University.

* Corresponding author.

** Corresponding author.

***Corresponding author.

E-mail addresses: rutianli@nju.edu.cn (Rutian Li), wangqiujing00@126.com (Qin Wang), baoruiliu@nju.edu.cn (Baorui Liu).

Table s1. Characterization of the NPs@GA. （*‾X±SD,n=3* ）

| Nanoparticles | Diameter (nm) | Zeta potential (Mv) | Polydispersity index |
| --- | --- | --- | --- |
| NPs@GA | 173.2±1.19 | -21.7±1.46 | 0.057±0.01 |

Table s2. Encapsulation efficiency and drug-loading capacity of the NPs@GA.（*‾X±SD,n=3* ）

| GA（mg） | mPEG-pep-PCL copolymer (mg) | Encapsulation efficiency（%） | Drug loading capacity（%） |
| --- | --- | --- | --- |
| 2mg | 4mg | 83.05%±0.02 | 25.31%±0.03 |


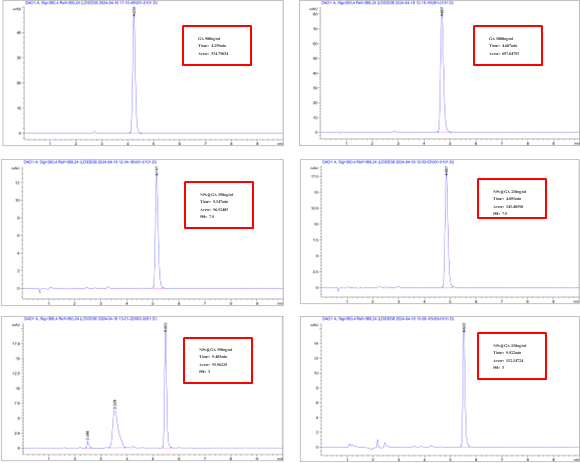


Fig.s1. The peak retention time of GA and NPs@GA using high-performance liquid chromatography (HPLC).


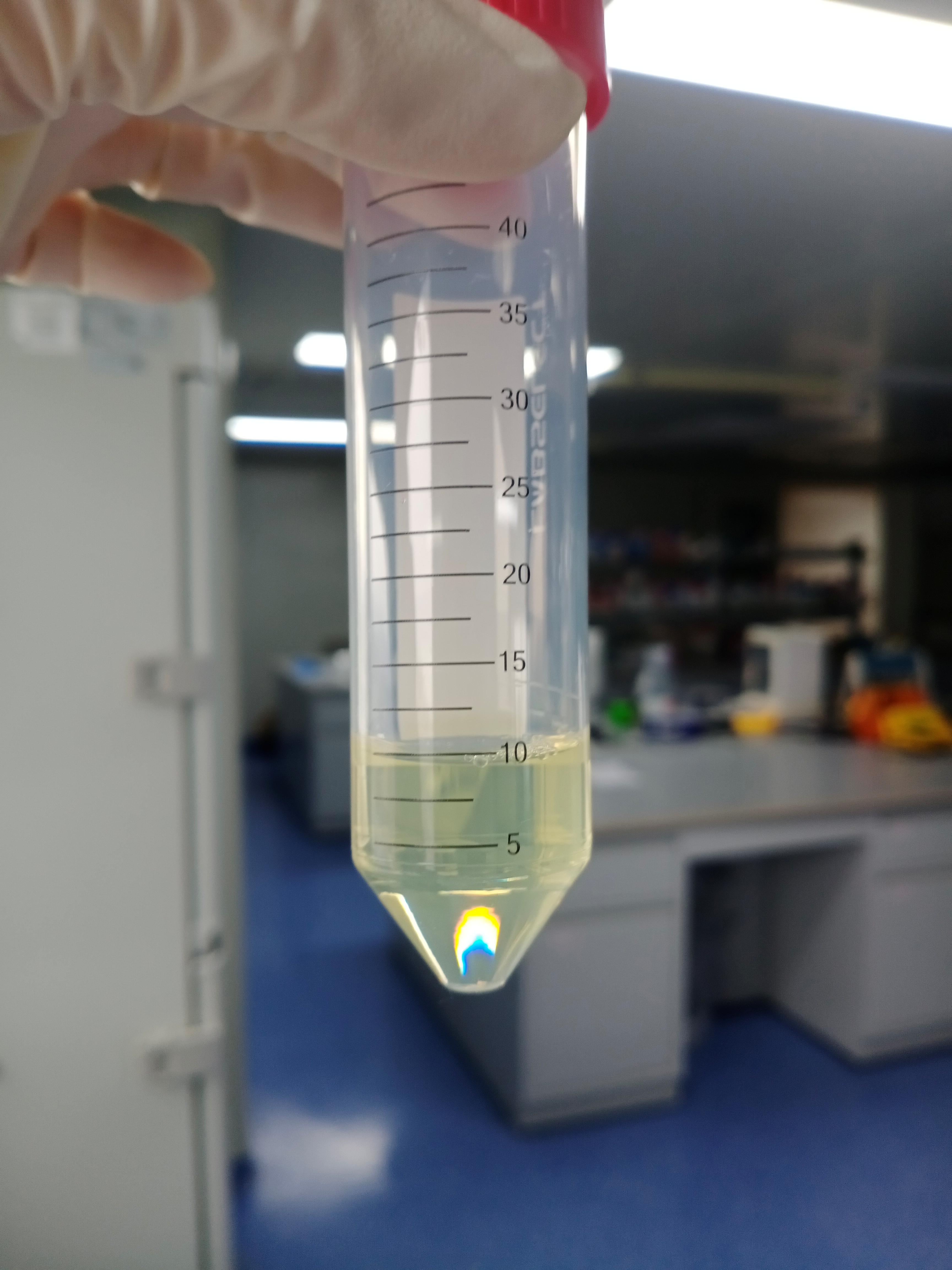

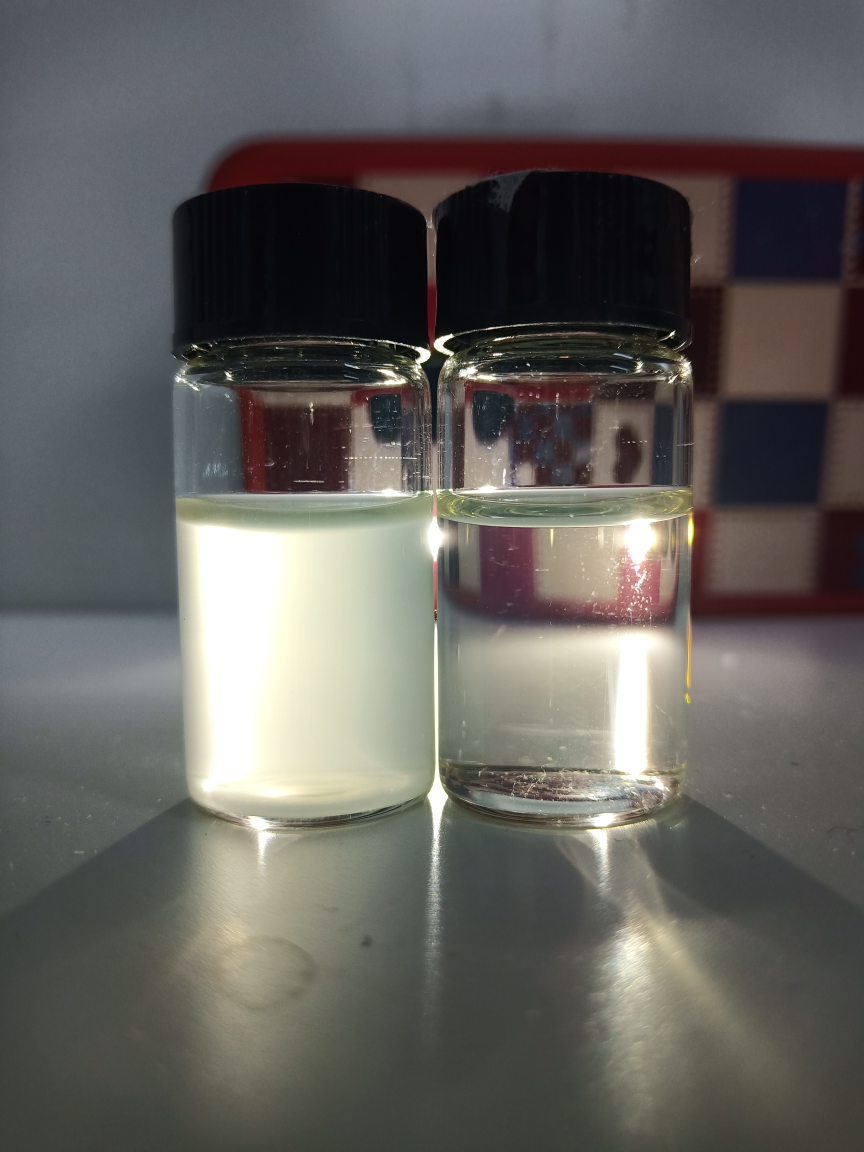


(a)

(b)

①

②

Fig.s2. In vitro targeting experiments of NPs@GA. (a. Clarified gambogic acid Nanoparticle Solution. b-c. ①and② are solutions of gambogic acid nanoparticles with and without collagenase, respectively.)


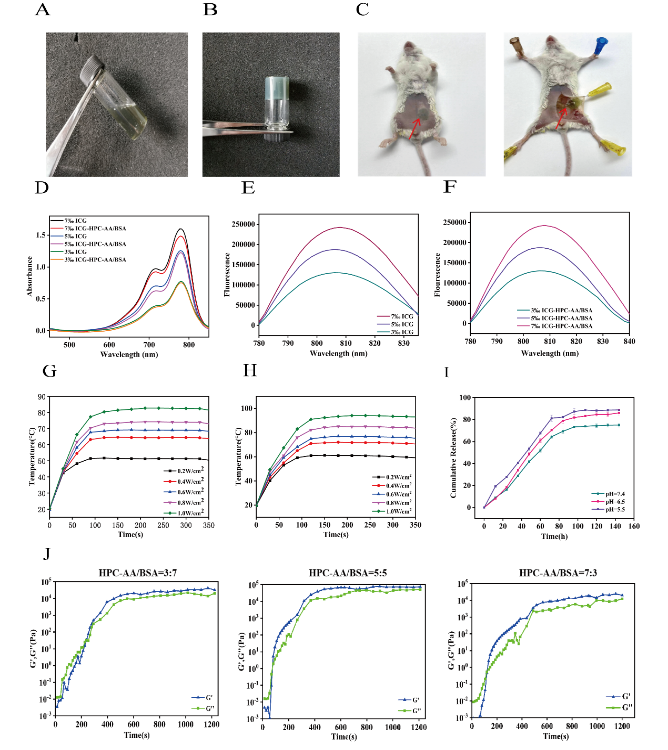

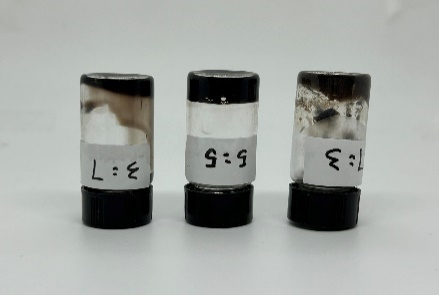

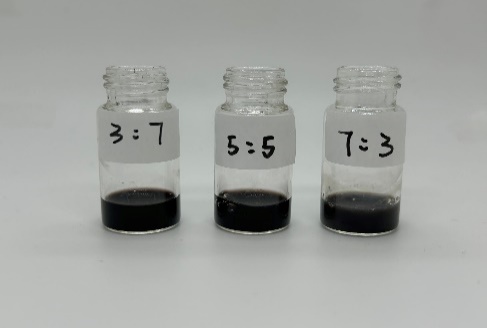
Fig. s3. In vitro sol-gel conversion experiments (a. Hydrogel solutions in different proportions. b. Gel formation of different ratios of hydrogels after heating in a water bath at 37 degrees Celsius for 4 minutes.).

(b)

(a)


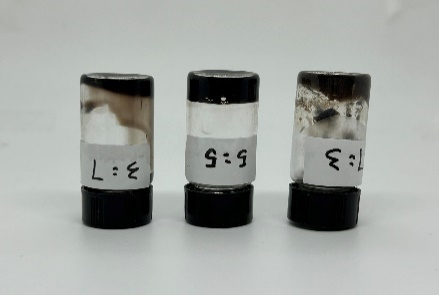

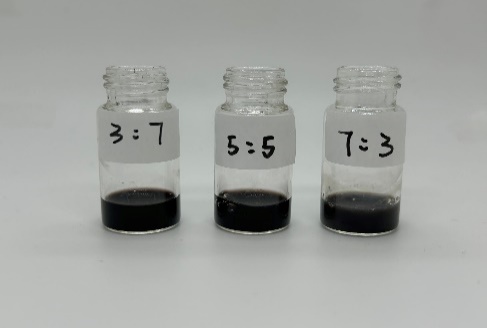


Fig. s4. Determination of rheological properties of HPC-AA/BSA hydrogel with different proportions(1).


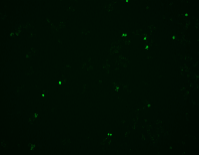

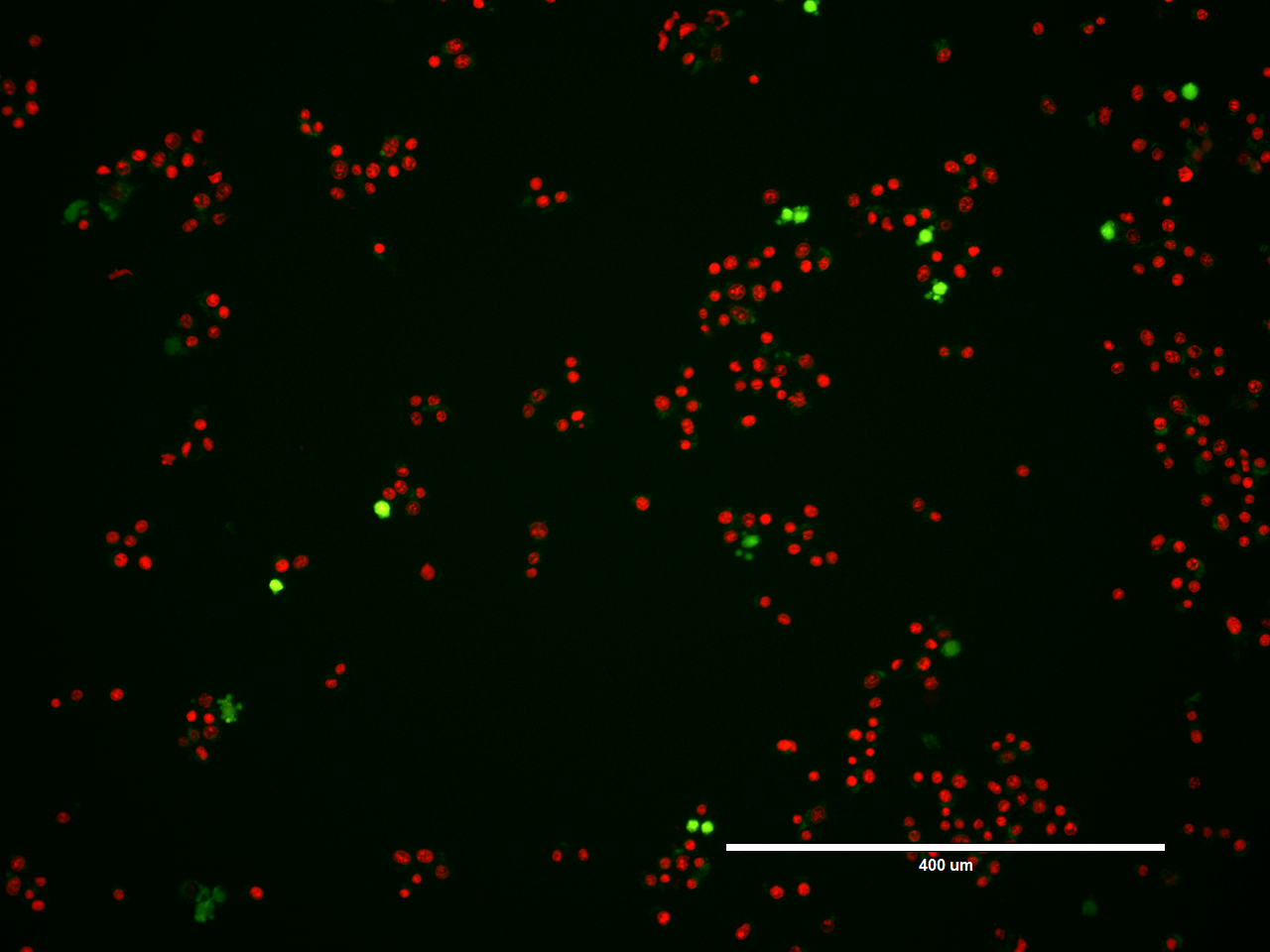

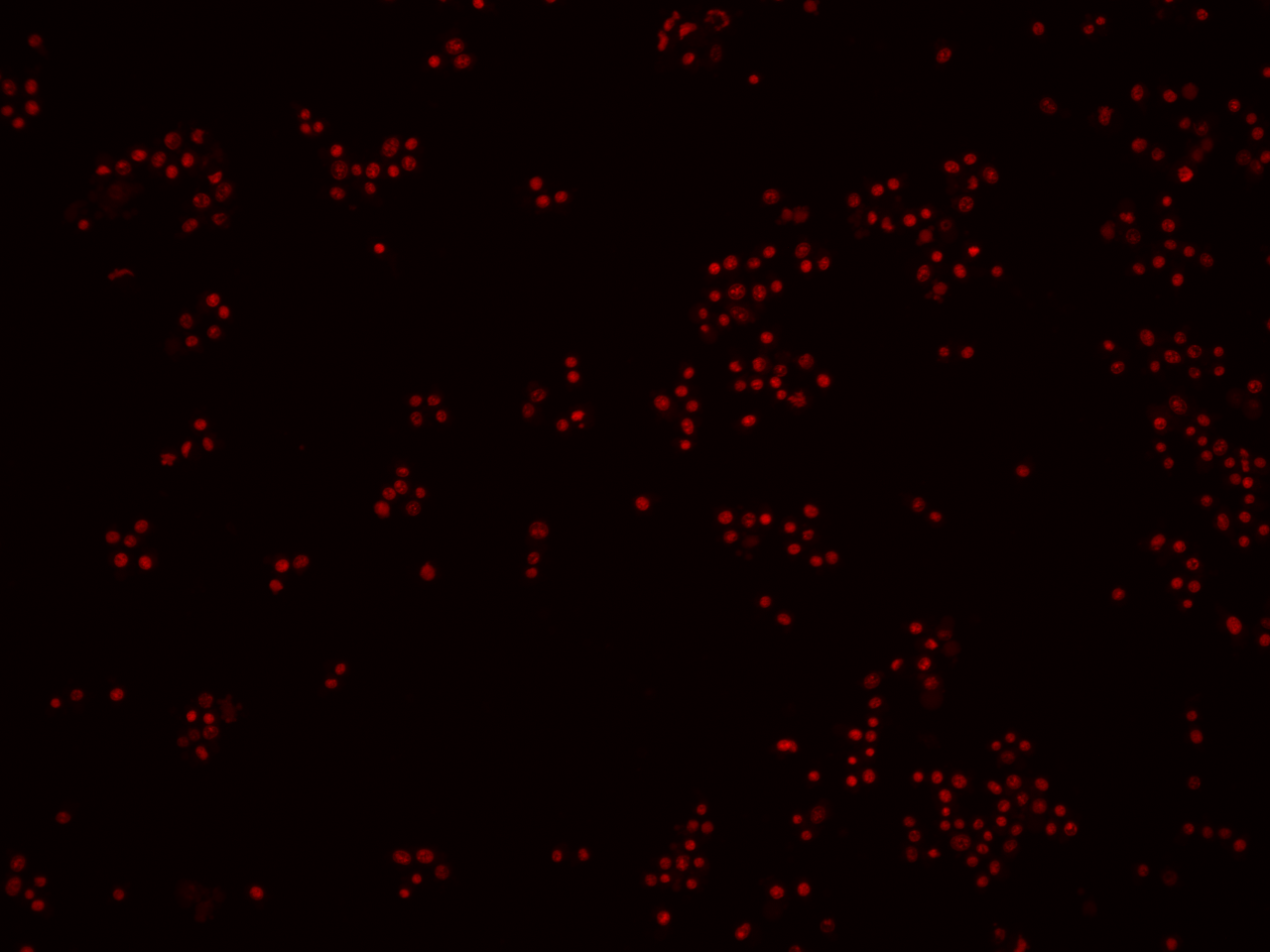

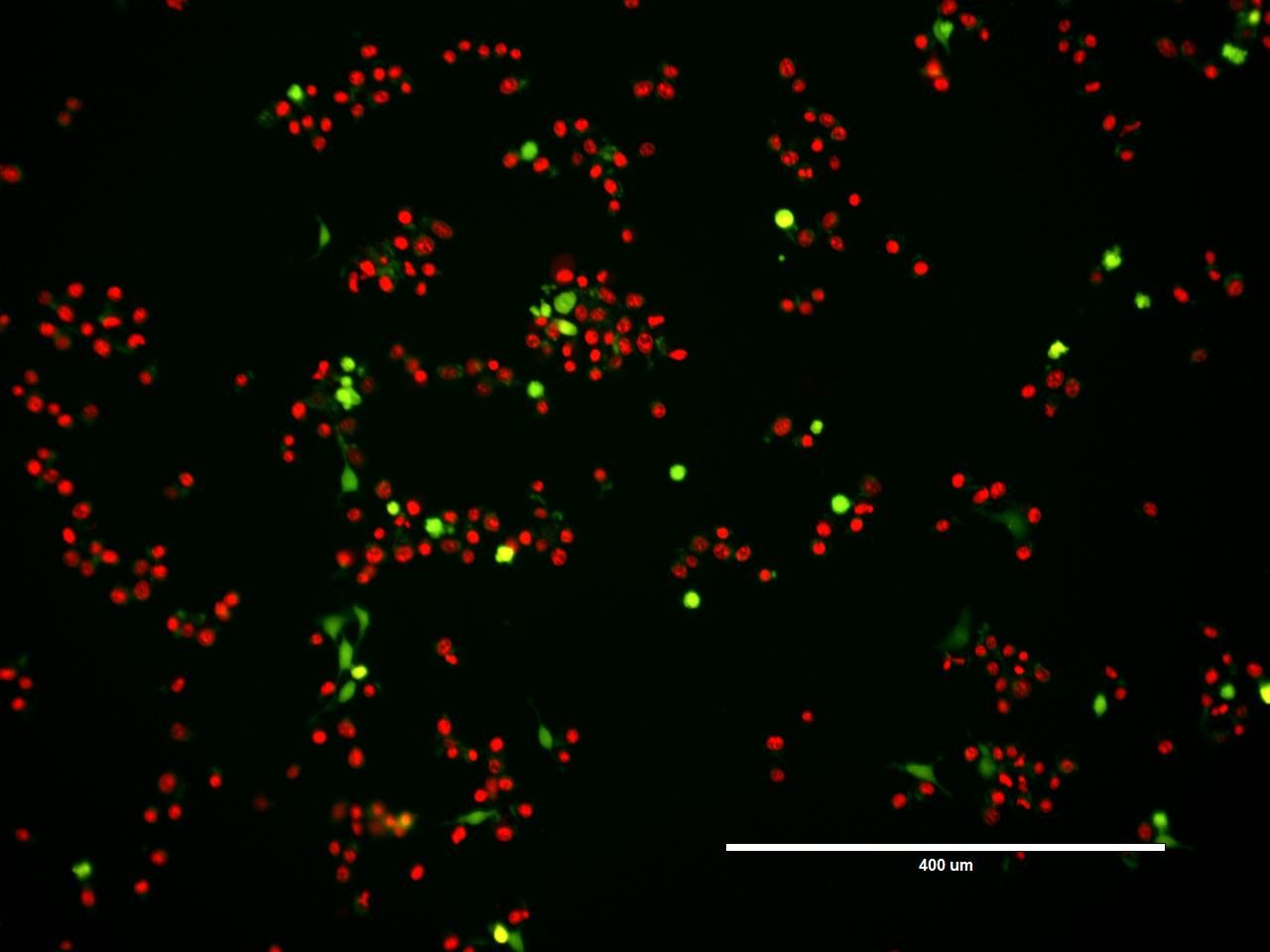

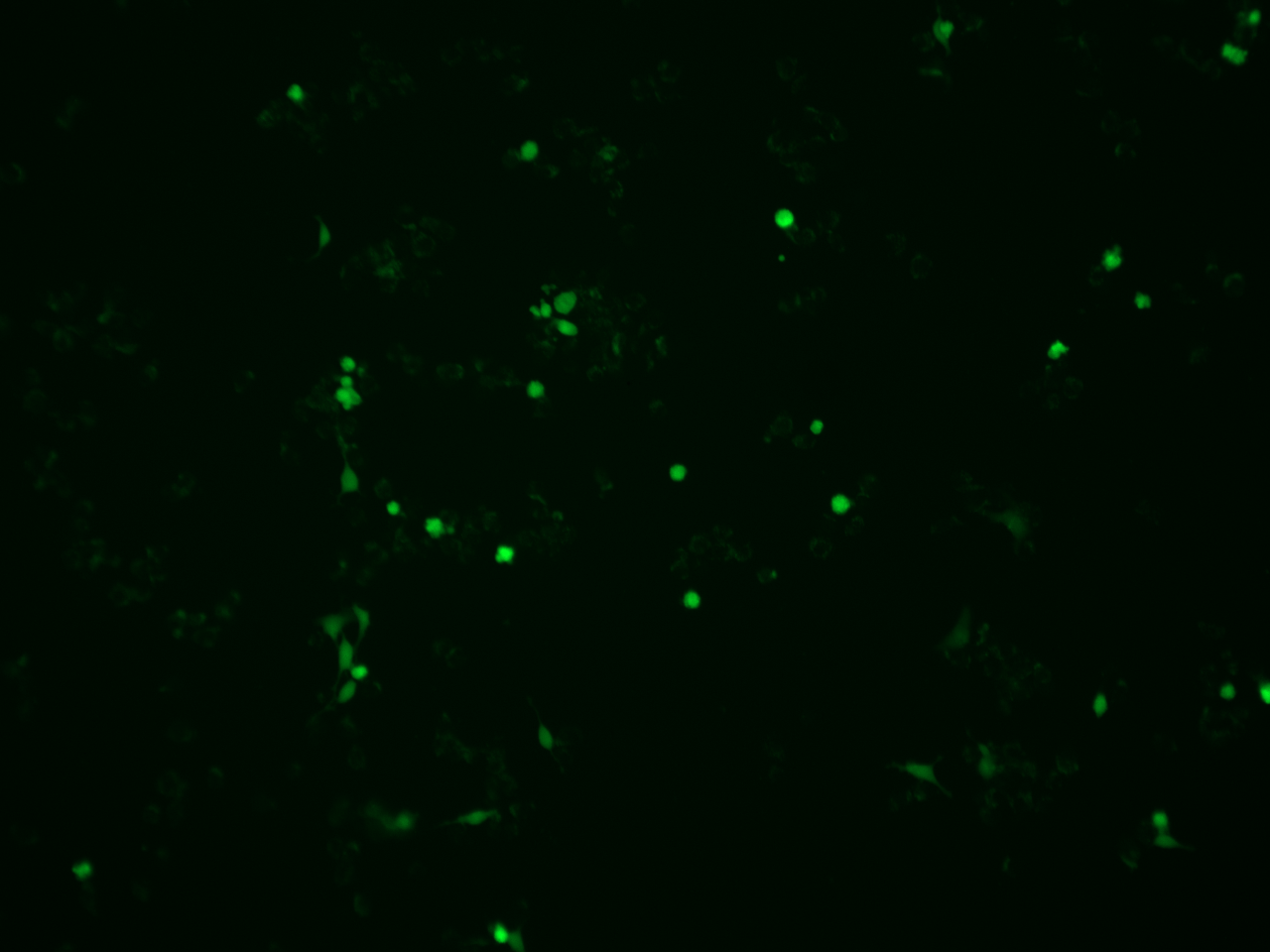

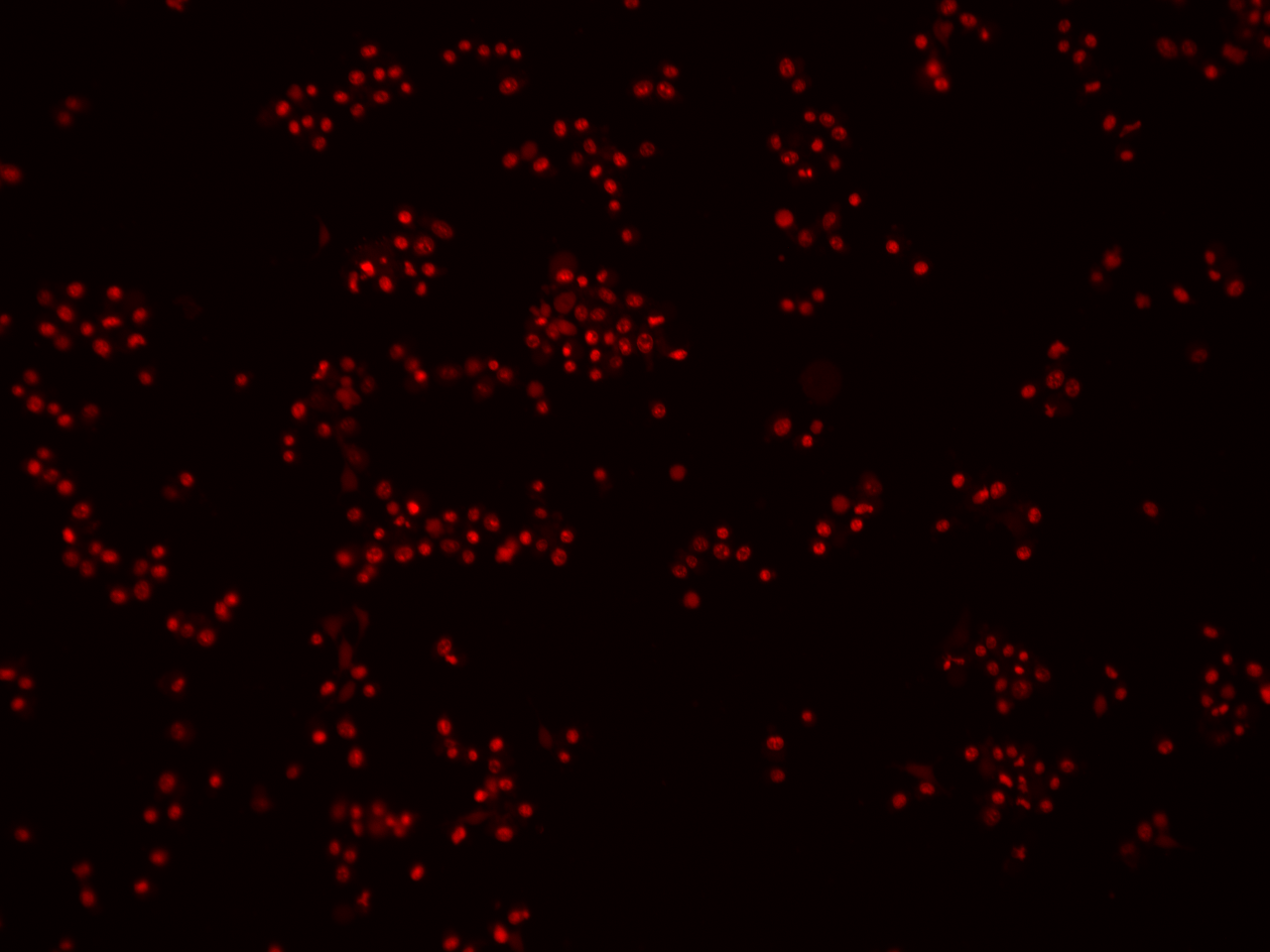


GA-NP

GA-NP@Gel

Merge

PI

AM

NS


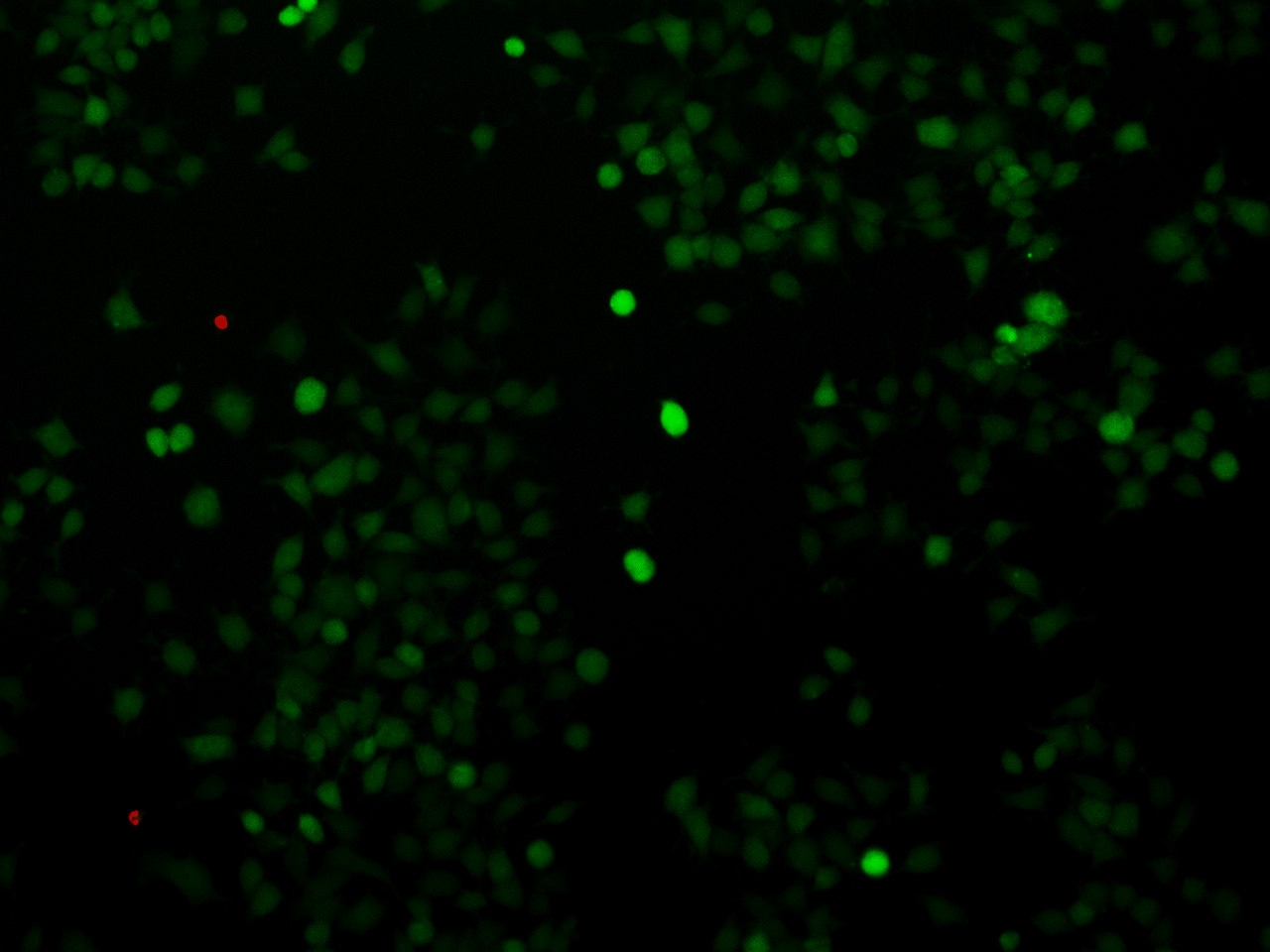

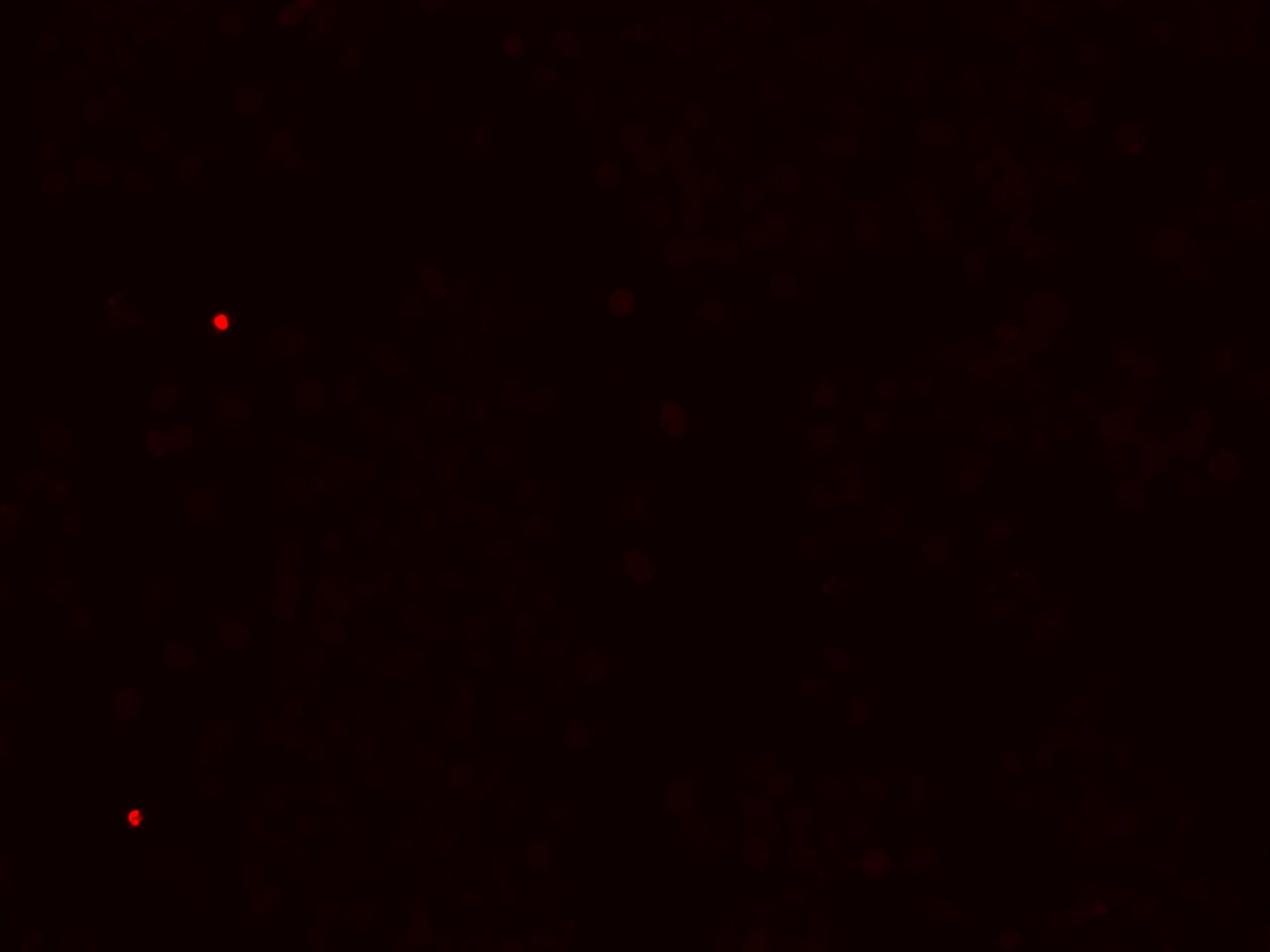

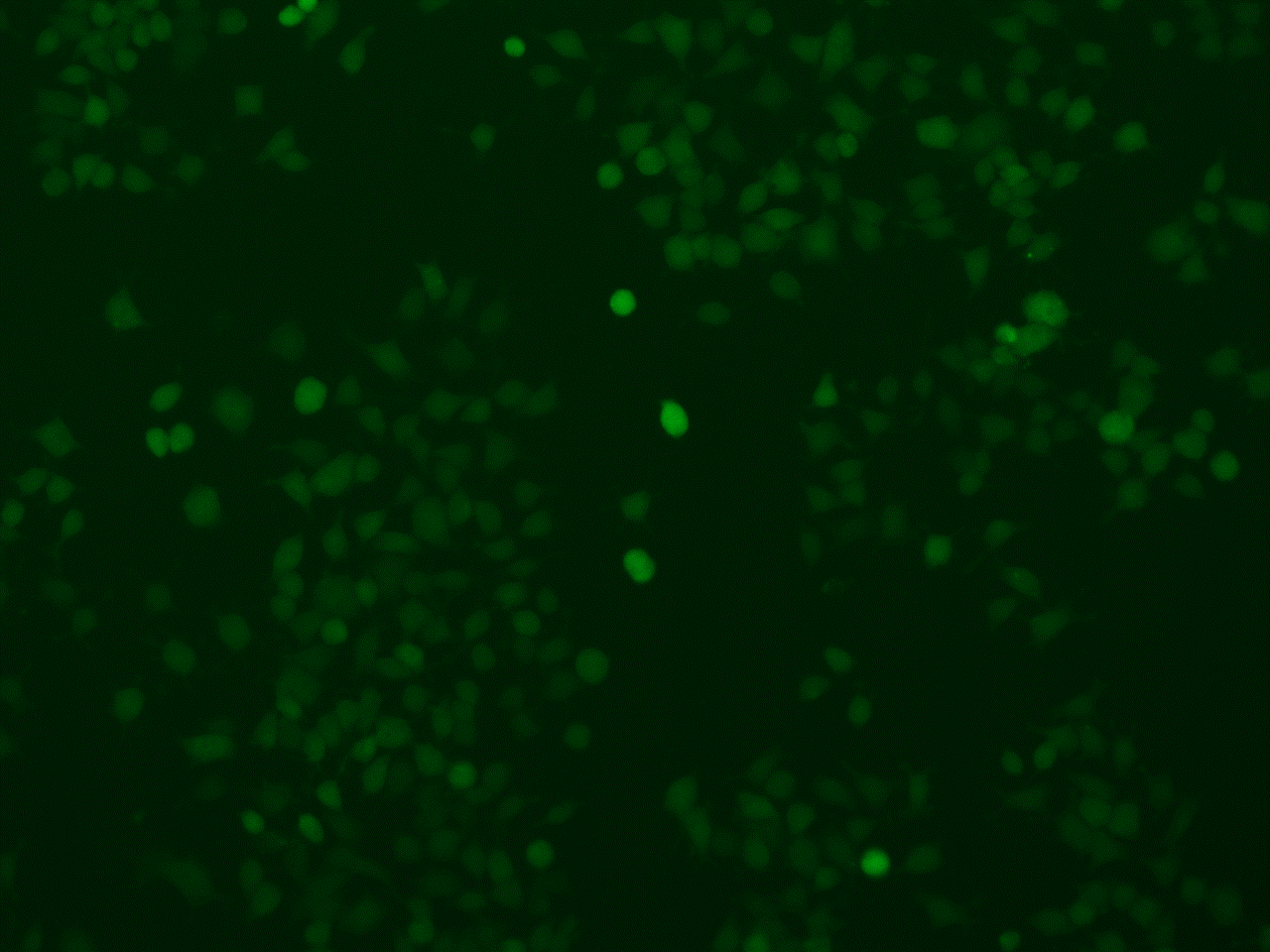


Fig. s5. The representative fluorescent images of in vitro cytotoxicity of different concentrations of Gel-NPs@GA, NPs@GA and GA acting on CT26.

**References:**

1. Li C, Lei D, Huang Y, Jing Y, Wang W, Cen L, et al. Remodeling the tumor immune microenvironment through hydrogel encapsulated G-Rh2 *in situ* vaccine and systemic immunotherapy. Materials Today Bio. 2024 Dec 1;29:101281.
